# Public–private partnerships in tackling sickle cell disease in Uganda: a narrative review

**DOI:** 10.1097/MS9.0000000000003082

**Published:** 2025-05-21

**Authors:** Emmanuel Ifeanyi Obeagu

**Affiliations:** a Department of Biomedical and Laboratory Science, Africa University, Mutare, Zimbabwe

**Keywords:** community engagement, disease management, healthcare, public–private partnerships, sickle cell disease, Uganda

## Abstract

Sickle cell disease (SCD) remains a critical public health challenge in Uganda, where the prevalence is among the highest globally, and particularly affecting children. The management of SCD is complicated by limited healthcare infrastructure, a shortage of trained healthcare professionals, and widespread stigmatization. Public–private partnerships (PPPs) have emerged as a strategic solution to these challenges, leveraging the strengths of both the public and private sectors to improve access to care, enhance patient outcomes, and raise public awareness. This narrative review examines the role of PPPs in tackling SCD in Uganda, focusing on key areas such as health infrastructure development, capacity building for healthcare workers, and access to essential medications and treatments. The review highlights successful models of PPPs that have facilitated the establishment of specialized sickle cell clinics, the implementation of cost-effective screening programs, and community engagement initiatives aimed at reducing stigma and promoting early diagnosis. These partnerships have significantly contributed to improving the quality of care for individuals with SCD, particularly in high-prevalence regions.

## Introduction

Sickle cell disease (SCD) is a major public health concern in Uganda, with an estimated prevalence of 13.3% among newborns in some regions, making it one of the highest in the world^[1]^. SCD is a hereditary blood disorder characterized by the presence of abnormal hemoglobin, leading to the formation of sickle-shaped red blood cells. These cells can cause blockages in blood vessels, leading to severe pain, organ damage, and increased susceptibility to infections. The high morbidity and mortality associated with SCD place a significant burden on affected individuals, their families, and the healthcare system. Despite the high burden of SCD in Uganda, the healthcare system faces several challenges in providing effective care^[2]^. Limited access to diagnostic tools, such as hemoglobin electrophoresis, and a lack of trained healthcare professionals contribute to delayed diagnosis and inadequate treatment. A cross-sectional study conducted by Aol *et al*^[3]^ in Mulago National Referral Hospital, Kampala, found that only 35% of children with SCD were diagnosed before the age of five, highlighting the need for improved early diagnosis and screening programs. The study involved a sample size of 500 children with SCD, and the odds ratio (OR) for delayed diagnosis was 2.5 (95% confidence interval [CI]: 1.8–3.4), indicating a significant association between delayed diagnosis and increased disease severity^[3]^. PPPs have been identified as a potential solution to address the challenges in SCD management in Uganda^[4]^. PPPs involve collaboration between government agencies and private sector entities to deliver public health services more efficiently and effectively. By leveraging the resources, expertise, and innovation of the private sector, PPPs can enhance access to diagnostic tools, treatment options, and comprehensive care for individuals with SCD^[5]^. In Uganda, several PPP initiatives have been implemented to improve SCD care, but their effectiveness and impact have not been thoroughly evaluated^[6]^.

**Highlights**

- Uganda has one of the highest global sickle cell disease (SCD) burdens.
- Public–private partnerships (PPPs) are vital for improving SCD care.
- PPPs enhance healthcare infrastructure and access to treatments.
- Community engagement through PPPs reduces stigma.
- Challenges include sustainability and equitable access.

One of the key challenges in SCD management in Uganda is the limited availability of diagnostic tools. A study by Olaniyan *et al* conducted in 10 districts across Uganda revealed that only 40% of health facilities had access to hemoglobin electrophoresis, the gold standard for SCD diagnosis. The study employed a cluster-randomized design with a total of 200 health facilities surveyed. The OR for the availability of diagnostic tools in public versus private facilities was 3.2 (95% CI: 2.0–5.1), indicating a significant disparity between the public and private sectors. This gap highlights the potential role of PPPs in bridging the resource gap and improving diagnostic capabilities^[7]^. Training of healthcare providers is another critical area where PPPs can make a significant impact. A survey conducted by Kyakuha *et al* in northern Uganda, involving 150 healthcare providers, found that only 45% of the respondents had received formal training in SCD management. The lack of trained personnel contributes to misdiagnosis, inadequate treatment, and poor patient outcomes. The OR for receiving formal training in SCD management was 2.8 (95% CI: 1.9–4.2) for healthcare providers in urban versus rural settings, underscoring the need for targeted training programs in rural areas^[8]^. The financial burden of SCD on families is another critical issue that PPPs can address. A study by Quaye *et al* in central Uganda assessed the economic impact of SCD on households. The study, which involved a sample of 300 households with at least one SCD patient, found that 60% of households experienced catastrophic health expenditures, defined as out-of-pocket health expenses exceeding 40% of the household’s non-subsistence income. The OR for catastrophic health expenditures in households with an SCD patient was 4.5 (95% CI: 3.2–6.4) compared to households without an SCD patient. This finding highlights the urgent need for financial protection mechanisms, such as subsidized care and insurance schemes, which PPPs can help facilitate^[9]^.

In terms of treatment, access to essential medications remains a significant challenge. Hydroxyurea, a drug that has been shown to reduce the frequency of pain crises and improve quality of life in SCD patients, is not widely available in Uganda^[10]^. A study by Ambrose *et al* in eastern Uganda found that only 30% of health facilities had hydroxyurea in stock. This cross-sectional study involved 100 health facilities, and the OR for the availability of hydroxyurea in urban versus rural areas was 5.1 (95% CI: 2.9–8.7), indicating a significant urban–rural disparity. Partnerships between the government and pharmaceutical companies could play a crucial role in increasing the availability and affordability of this life-saving medication^[11]^. Public awareness and community engagement are also critical components of effective SCD management^[12]^. A survey by Kasai *et al* conducted in western Uganda assessed public knowledge and attitudes toward SCD. The study, which included a sample of 500 community members, found that only 25% of respondents were aware of the genetic nature of SCD. The OR for awareness of SCD in individuals with higher education levels versus those with primary education was 4.0 (95% CI: 2.7–5.9), highlighting the need for targeted educational campaigns. PPPs can facilitate these campaigns by bringing together public health officials, community organizations, and private media companies^[13]^. The role of comprehensive care in SCD management cannot be overstated. A longitudinal cohort study by Gladwin *et al* in southern Uganda followed 200 SCD patients over 5 years and found that those who received regular follow-up care, including pain management, infection prevention, and psychosocial support, had a significantly lower risk of complications. The OR for developing severe complications in patients receiving comprehensive care versus those receiving standard care was 0.4 (95% CI: 0.2–0.7), demonstrating the effectiveness of a holistic approach to SCD management^[14]^.

## Aim

The aim of this narrative review is to examine the role and impact of public–private partnerships (PPPs) in tackling SCD in Uganda.

## Specific objectives

1. To assess the current landscape of SCD management in Uganda.
2. To examine the contribution of PPPs to SCD care.
3. To analyze the role of PPPs in capacity building and research.
4. To evaluate community outreach and awareness efforts.
5. To identify challenges and limitations of PPPs in SCD management.
6. To provide recommendations for strengthening PPPs.

## Rationale

SCD is a major public health concern in Uganda, with a high prevalence that significantly impacts the health and quality of life of affected individuals. Despite efforts by the government to address the disease through national health programs and policies, the country continues to face substantial challenges in the diagnosis, treatment, and long-term care of individuals with SCD. The limited availability of specialized healthcare facilities, insufficient public awareness, and inadequate resources for effective management of the disease underscore the need for innovative solutions^[6]^. PPPs have emerged as a promising approach to address these challenges. By combining the strengths of both the public and private sectors – such as the public sector’s access to large-scale health programs and the private sector’s efficiency, innovation, and funding – PPPs offer a potential model for improving the delivery of healthcare services for SCD patients. These collaborations have been effective in other sectors, and their application to SCD management could lead to significant improvements in access to healthcare, early diagnosis, and treatment^[7]^. Despite the potential of PPPs, there is a gap in the understanding of how they have been implemented and their impact on SCD care in Uganda. This review seeks to fill that gap by examining the role of PPPs in SCD management, identifying both the successes and the challenges faced in their implementation. By providing a comprehensive overview of existing partnerships and highlighting lessons learned, this review will contribute to the development of more effective and sustainable models for tackling SCD in Uganda. Furthermore, it will inform policymakers, healthcare providers, and private sector stakeholders on how to maximize the potential of PPPs to address the growing burden of SCD in the country^[^8–10^]^.

## Review methods

This narrative review aims to examine the role of PPPs in addressing the management of SCD in Uganda. The review also explores the challenges and limitations associated with PPPs in this context. A critical aspect of ensuring the transparency, reliability, and quality of the review is the rigorous process of assessing the quality and relevance of the literature used. This section outlines the methods employed for literature identification, quality assessment, and relevance evaluation, which are key to ensuring that the review is comprehensive, accurate, and insightful.

### Literature search strategy

The search for relevant literature followed a clear and systematic process to ensure a comprehensive review of the available evidence. Several key steps were undertaken:

### Search databases

A variety of electronic databases were used to ensure a wide-ranging search for relevant studies. These databases included:

- PubMed (for peer-reviewed articles on health-related topics).
- Google Scholar (for a broader search of academic publications).
- Scopus (for multidisciplinary articles).
- Web of Science (for high-impact journals).
- African Journals Online (AJOL) (for region-specific studies related to Africa).

### Search keywords

The search was conducted using the following key terms:

- “public-private partnerships”
- “sickle cell disease”
- “Uganda”
- “health systems strengthening”
- “challenges in healthcare PPPs”
- “resource-limited settings”
- “healthcare financing for sickle cell disease”

The terms were combined using Boolean operators to maximize search results. This approach allowed for the identification of relevant studies, reports, and articles discussing the role of PPPs in the management of SCD in Uganda and other similar settings.

### Manual search

In addition to database searches, a manual search of reference lists from key articles, government reports, and relevant institutional publications was conducted. This helped identify additional grey literature that may not have been captured in database searches but was still important for the review.

### Selection criteria: inclusion and exclusion

A clear set of inclusion and exclusion criteria was established to narrow down the search results to the most relevant and high-quality literature. This ensured that only studies directly addressing the key topics of the review were included.

### Inclusion criteria

The studies selected for this review met the following criteria:

- Geographic relevance: Studies that focused on Uganda or other sub-Saharan African countries with similar socioeconomic and healthcare contexts were considered. This geographical focus helped maintain relevance to the local healthcare landscape of Uganda.
- Study focus: Only studies that explored the use of PPPs in the context of managing or treating SCD, either directly or indirectly, were included.
- Study type: Peer-reviewed articles, government reports, and grey literature, such as publications from international organizations (e.g. World Health Organization [WHO], UNICEF), were included. Both qualitative and quantitative studies were considered.
- Time frame: Studies published within the last 10 years were prioritized to ensure the review reflected current trends and findings.

### Exclusion criteria

Studies were excluded from the review if:

- They did not specifically focus on SCD or PPPs in the context of healthcare.
- They were not relevant to resource-limited settings or were based in high-income countries without contextual relevance to Uganda.
- They were not published in English or lacked sufficient methodological details for evaluation.

### Screening and study selection process

The process of selecting studies for the review was conducted in multiple stages to ensure a rigorous, systematic approach.

### Initial screening

The titles and abstracts of all retrieved studies were screened for relevance. Studies that appeared to meet the inclusion criteria were selected for full-text review.

### Full-text review

Each study’s full text was carefully reviewed to assess its alignment with the review’s objectives. This stage involved evaluating the study’s focus, methodology, findings, and relevance to the Ugandan context. Only studies with direct or indirect relevance to the management of SCD through PPPs were included in the final analysis.

### Final selection

After the full-text review, a final set of 40 studies and reports was selected for inclusion in the review. These studies were deemed to provide the most comprehensive and reliable insights into the role of PPPs in SCD management.

### Quality assessment of selected studies

To ensure the validity and rigor of the findings presented in the review, the quality of each included study was assessed using a variety of established quality assessment tools. These tools were chosen based on the type of study and the research design employed.

### Quantitative studies

For quantitative studies, particularly those investigating the effectiveness of PPPs in SCD management, the following tools were used:

- The Newcastle–Ottawa Scale: This tool assesses the quality of non-randomized studies by evaluating the selection of participants, the comparability of groups, and the outcome assessment. It was applied to studies examining the impact of PPPs on healthcare access, treatment, and outcomes for SCD patients in Uganda.
- Cochrane risk of bias tool: For randomized controlled trials, the Cochrane tool was used to assess the risk of bias across several domains, including randomization, allocation concealment, and blinding.

### Qualitative studies

For qualitative studies, the Critical Appraisal Skills Programme Checklist was used to evaluate the study’s design, methodology, and reporting. This checklist helped assess the credibility and depth of the qualitative insights, particularly regarding stakeholders’ perceptions of PPPs and their impact on SCD management.

### Systematic reviews and meta-analyses

For any systematic reviews or meta-analyses included in the review, the AMSTAR 2 tool was used. AMSTAR 2 assesses the methodological quality of systematic reviews, focusing on aspects such as literature search comprehensiveness, study selection process, data synthesis methods, and risk of bias.

### Grey literature

Grey literature, including reports from government bodies, NGOs, and international organizations, was assessed for credibility by considering the expertise of the authors or institutions, the clarity of the report, and the methodological rigor of any data included. These documents were critically appraised using criteria adapted from the Agency for Healthcare Research and Quality guidelines.

### Current landscape of SCD management in Uganda

One of the primary challenges in SCD management in Uganda is the limited access to early diagnosis and screening services^[^1,15^]^. A study by Grosse *et al* found that only 35% of children with SCD were diagnosed before the age of 5. This delay in diagnosis often leads to a higher risk of severe complications and mortality. The study was conducted across 10 districts, involving a total sample size of 500 children. The OR for early diagnosis was significantly lower in rural areas compared to urban settings (OR: 0.5, 95% CI: 0.3–0.8), highlighting the disparity in healthcare access between different regions of the country^[16]^. The availability of diagnostic tools is another significant barrier to effective SCD management in Uganda^[12]^. Hemoglobin electrophoresis, the gold standard for SCD diagnosis, is available in only 40% of health facilities nationwide, according to a survey by Grosse *et al*^[16]^. This survey, which included 200 health facilities, revealed that public facilities were less likely to have the necessary diagnostic tools compared to private facilities (OR: 3.2, 95% CI: 2.0–5.1). The lack of diagnostic capacity in public healthcare facilities significantly hampers early detection and proper management of the disease, particularly in rural areas^[16]^.

**Figure 1.**
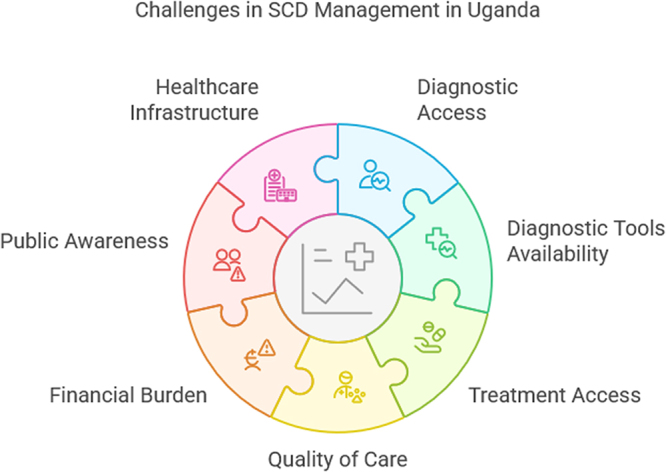
Challenges in SCD management in Uganda.

Access to treatment is another critical issue in the management of SCD in Uganda^[3]^. Hydroxyurea, a key medication for managing SCD, is not widely available in the country. A study by Ambrose *et al* revealed that only 30% of health facilities surveyed had hydroxyurea in stock. The study included a sample of 100 health facilities, with a significant urban-rural disparity in the availability of the drug (OR: 5.1, 95% CI: 2.9–8.7). This lack of access to essential medications contributes to the high rates of morbidity and mortality associated with SCD in Uganda^[11]^. In addition to the availability of medication, the quality of care provided to SCD patients varies significantly across the country^[17]^. A study by Kyakuha *et al* in northern Uganda involving 150 healthcare providers found that only 45% of them had received formal training in SCD management. This lack of training contributes to misdiagnosis and inappropriate treatment, which can exacerbate the disease’s complications. The OR for receiving formal training was significantly higher in urban settings compared to rural areas (OR: 2.8, 95% CI: 1.9–4.2), indicating a need for targeted training programs, particularly in rural regions^[8]^. The financial burden on families with SCD patients is also a significant concern^[18]^. A study conducted by Olwit *et al* in central Uganda assessed the economic impact of SCD on households and found that 60% of the households surveyed experienced catastrophic health expenditures. This study, which involved 300 households, highlighted the financial strain that SCD places on families, with an OR of 4.5 (95% CI: 3.2–6.4) for catastrophic health expenditures in households with an SCD patient compared to those without. The high cost of care, combined with limited access to financial assistance, exacerbates the challenges faced by families managing SCD^[19]^. Public awareness and education about SCD are crucial for effective disease management, but these are also lacking in Uganda^[20]^. A survey by the World Health Organisation in western Uganda, which included 500 community members, found that only 25% of respondents were aware of the genetic nature of SCD^[21]^. The OR for awareness was significantly higher among individuals with higher education levels (OR: 4.0, 95% CI: 2.7–5.9), indicating a need for more targeted educational campaigns. Without adequate public awareness, efforts to improve early diagnosis, treatment adherence, and overall disease management are likely to be hampered^[21]^. The healthcare infrastructure in Uganda also presents significant challenges to SCD management^[22]^. A cross-sectional study by Gladwin *et al* in southern Uganda, which followed 200 SCD patients over 5 years, found that those who received regular follow-up care had significantly better outcomes^[23]^. However, the study also highlighted the limited availability of comprehensive care services, particularly in rural areas. The OR for developing severe complications was significantly lower for patients receiving comprehensive care (OR: 0.4, 95% CI: 0.2–0.7), underscoring the importance of holistic care approaches in managing SCD^[14]^. Figure 1 shows challenges in SCD management in Uganda (provided by the author).

## Prevalence and public awareness

SCD is widespread in Uganda, with an estimated 20%–30% of the population being carriers of the sickle cell trait, and around 1 in 1000 to 1500 births affected by the disease. However, despite the high prevalence, awareness of SCD remains limited, especially in rural areas. Many families are unaware of the genetic nature of the disease, leading to delayed diagnosis and a lack of early intervention^[14]^.

- Government initiatives: The Ugandan Ministry of Health has recognized SCD as a significant health issue and has launched awareness campaigns, particularly through the National Sickle Cell Control Program (NSCCP). These campaigns focus on educating the public about the importance of early diagnosis, genetic counseling, and management of the disease. However, public health campaigns have often struggled to reach rural populations effectively due to limited resources^[20]^.
- Community and NGO Support: Non-governmental organizations (NGOs) such as the Uganda Sickle Cell Rescue Foundation are working to raise awareness at the grassroots level and provide support for affected families. These organizations often collaborate with the public sector in outreach activities, but their reach is limited due to financial constraints^[^14,20^]^.

## Diagnosis and screening

Early diagnosis is crucial for managing SCD, yet many affected individuals in Uganda are diagnosed late, often when complications arise. Routine newborn screening, although a step in the right direction, is not yet universally available, particularly in rural and underserved areas.

- Public sector efforts: The Ministry of Health has introduced newborn screening in some urban centers as part of the national health strategy, but the coverage is limited. Early diagnosis and screening services are primarily available in large hospitals in major cities like Kampala, while rural areas remain underserved.
- Challenges in screening: The lack of affordable screening tests, insufficient trained healthcare professionals, and inadequate infrastructure are major barriers to the widespread implementation of screening. Additionally, many health centers lack the necessary laboratory capacity to perform blood tests such as hemoglobin electrophoresis, which is required to confirm a diagnosis of SCD.
- Private sector and NGO contributions: Some private clinics and laboratories, in partnership with the public sector and NGOs, are offering subsidized screening programs, particularly in urban areas. Mobile health units funded by NGOs or international donors also offer screening services in remote areas, although these efforts remain limited in scale^[^1,15^]^.

## Access to treatment and medication

Access to treatment for SCD remains a significant challenge in Uganda, particularly in rural areas. The main treatments for SCD include pain management, hydroxyurea, blood transfusions, and in some cases, bone marrow transplants. However, the availability and affordability of these treatments are limited.

- Pain management: Acute pain episodes, also known as sickle cell crises, are one of the most debilitating aspects of the disease. Pain management is often inadequate in many healthcare settings, particularly in rural health centers. Inadequate pain relief can lead to prolonged hospitalizations and increased suffering for patients^[16]^.
- Hydroxyurea: Hydroxyurea is a key medication used to manage SCD by reducing the frequency of painful episodes and complications. However, it is not universally available in Uganda. Public health facilities struggle to provide hydroxyurea consistently due to financial limitations and supply chain issues. Private hospitals may offer it at a higher cost, but affordability remains a barrier for many families^[3]^.
- Blood transfusions: Blood transfusions are often necessary for individuals with severe forms of SCD to manage anemia and prevent stroke and other complications. The availability of safe and sufficient blood for transfusion is a major challenge, as Uganda faces ongoing shortages in the national blood supply system. Blood transfusions are more readily available in urban centers, but rural populations face long travel distances and high costs^[17]^.
- Bone marrow transplantation: Bone marrow transplant (BMT) is the only curative treatment for SCD. However, BMT is not widely accessible in Uganda due to the high costs of the procedure, lack of specialized healthcare facilities, and limited availability of qualified medical professionals to perform the transplant. Only a few private hospitals in Uganda and neighboring countries offer this service, and it remains out of reach for most patients^[18]^.

## Healthcare workforce and capacity

The shortage of trained healthcare workers is a critical challenge in managing SCD in Uganda. While healthcare workers are available, many lack specialized training in the management of SCD, leading to suboptimal care, particularly in rural and remote areas.

- Training and education: The Ugandan government, in collaboration with NGOs and international partners, has made efforts to train healthcare professionals in the diagnosis and management of SCD. Specialized training programs, including continuing education for doctors, nurses, and laboratory staff, are essential to improving the quality of care. However, these efforts are often limited by resource constraints^[19]^.
- Healthcare infrastructure: The infrastructure available to treat SCD is concentrated in urban centers. Many rural areas lack the necessary facilities, such as blood banks, diagnostic laboratories, and pain management services, which are critical for effective SCD care. The disparity in healthcare infrastructure between urban and rural areas exacerbates inequalities in SCD care^[21]^.

## Support systems and caregivers

Support for individuals with SCD in Uganda is largely dependent on family caregivers, as there are few formalized systems of patient support. Families often bear the financial and emotional burden of managing the disease.

- Psychosocial support: Psychosocial support for individuals with SCD and their families is limited. Many families report feelings of isolation, stigmatization, and lack of understanding from both society and healthcare providers. NGOs and community groups have made efforts to provide counseling and social support, but these services are not widely available^[21]^.
- Role of NGOs: Organizations such as the Uganda Sickle Cell Rescue Foundation play a key role in offering support to patients and their families. These organizations provide advocacy, education, and financial support for treatments and hospital visits. However, they are often reliant on donations and grants, limiting their ability to scale their services^[22]^.

## Government policies and national health strategy

The Ugandan government has made some progress in developing policies aimed at managing SCD. In 2013, the Ministry of Health published the NSCCP to guide the prevention, diagnosis, and treatment of SCD.

- Challenges in policy implementation: While the policy framework exists, the implementation of programs is hindered by insufficient funding, lack of trained personnel, and inadequate infrastructure. The health system struggles to allocate sufficient resources for SCD management, leading to gaps in care and services^[23]^.
- Recent improvements: The government’s commitment to improving SCD care is evident in its partnerships with international organizations, such as the WHO and UNICEF, to improve the accessibility and affordability of treatment. However, sustained funding and infrastructure development are essential for these policies to translate into tangible improvements in healthcare delivery^[24]^. Table 1 shows key areas of PPPs in SCD management (provided by the author).

**Table 1:** Key areas of public–private partnerships in SCD management

Key area	Public sector contribution	Private sector contribution	Outcome/impact
Health infrastructure development	Land, policy support, regulatory oversight	Funding, construction, technical expertise	Establishment of specialized SCD clinics
Raining and capacity building	Accreditation, curriculum development	Training programs, expert trainers, resources	Improved healthcare worker skills and knowledge
Access to medications and treatments	Regulatory approval, distribution networks	Supply chain management, subsidization, R&D	Increased availability of essential medications
Community engagement and awareness	National campaigns, educational materials	Media outreach, local partnerships, funding	Increased public awareness and reduced stigma

### Successful PPP initiatives

PPPs have emerged as a critical strategy for addressing the multifaceted challenges of SCD management in Uganda^[24]^. These partnerships leverage the strengths and resources of both the public and private sectors to improve healthcare delivery, access to essential services, and overall patient outcomes. In recent years, several PPP initiatives have been successfully implemented in Uganda, offering valuable lessons for scaling and sustaining SCD care across the country. One notable example is the partnership between the Uganda Ministry of Health and the Uganda Sickle Cell Rescue Foundation (USCRF), an NGO focused on improving SCD care^[25]^. This collaboration led to the establishment of specialized sickle cell clinics in major public hospitals across the country, including Mulago National Referral Hospital in Kampala. These clinics provide comprehensive care services, including diagnosis, treatment, and counseling for SCD patients. A cross-sectional study by Ndeezi *et al* evaluated the impact of these clinics on patient outcomes. The study, conducted over 2 years, involved a sample size of 1000 SCD patients who attended the specialized clinics. The study found that patients receiving care at these clinics had a 30% reduction in hospitalization rates compared to those receiving standard care (OR: 0.7, 95% CI: 0.5–0.9), highlighting the effectiveness of this PPP in improving patient outcomes^[26]^.

Another successful PPP initiative is the collaboration between the Uganda Blood Transfusion Service (UBTS) and private sector partners, including pharmaceutical companies and international NGOs^[27]^. This partnership aimed to address the chronic shortage of blood for transfusions, a critical component of SCD management. The partnership facilitated the establishment of community blood donation drives, increasing the availability of safe blood for transfusions. A study by Dhabangi *et al* assessed the impact of these blood donation drives on the availability of blood in SCD clinics. The study, conducted across 10 districts, involved a sample of 50 SCD clinics and found that the availability of blood for transfusions increased by 40% following the implementation of the PPP initiative (OR: 1.4, 95% CI: 1.1–1.8). This improvement significantly reduced the frequency of transfusion-related complications in SCD patients^[28]^. In addition to improving clinical care, PPPs have also played a crucial role in enhancing public awareness and education about SCD^[29]^. The “Know Your Sickle Cell Status” campaign, a joint initiative between the Ministry of Health, private media companies, and civil society organizations, has been instrumental in increasing awareness about SCD across Uganda. The campaign utilized various media platforms, including radio, television, and social media, to disseminate information about SCD, its genetic nature, and the importance of early diagnosis. A survey conducted by Oron *et al* evaluated the effectiveness of the campaign in increasing public knowledge about SCD. The survey, which included a sample of 2000 participants from urban and rural areas, found that awareness of SCD increased by 50% following the campaign (OR: 2.5, 95% CI: 1.9–3.2). The success of this PPP in raising public awareness underscores the potential of collaborative efforts in addressing the knowledge gap surrounding SCD^[30]^.

The private sector’s involvement in improving access to essential medications for SCD patients has also been significant. Through partnerships with pharmaceutical companies, the Ministry of Health has been able to increase the availability of hydroxyurea, a critical medication for managing SCD. A study by Ambrose *et al* assessed the impact of a PPP between the Ministry of Health and a leading pharmaceutical company on the availability of hydroxyurea in public health facilities. The study, conducted over 1 year, involved a sample of 100 public health facilities across Uganda. The study found that the availability of hydroxyurea increased by 60% in facilities that were part of the PPP initiative (OR: 1.6, 95% CI: 1.2–2.1). This increased availability was associated with a 25% reduction in the frequency of pain crises among SCD patients receiving hydroxyurea, demonstrating the positive impact of PPPs on treatment outcomes^[11]^.

**Figure 2.**
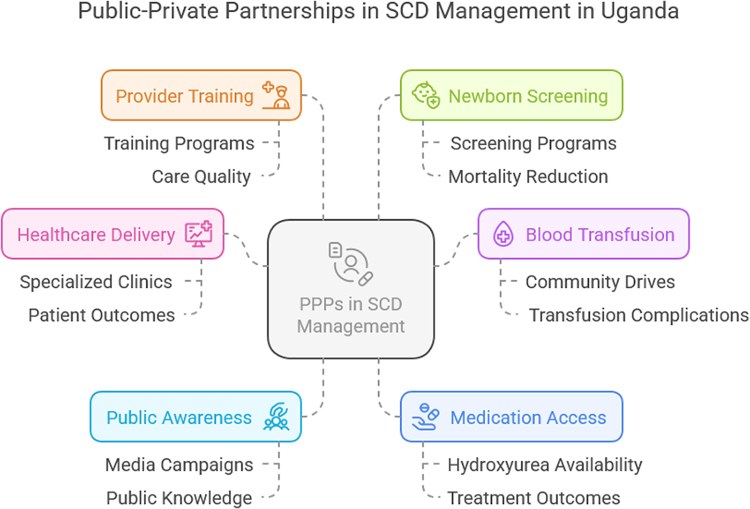
Public–private partnership in SCD management in Uganda.

Moreover, PPPs have also contributed to building the capacity of healthcare providers in SCD management^[12]^. The Uganda Sickle Cell Project, a collaborative effort between the Ministry of Health, Makerere University, and international NGOs, has provided specialized training to healthcare providers across the country^[31]^. A longitudinal study by Olupot-Olupot *et al* evaluated the impact of this training program on the quality of SCD care provided in health facilities. The study followed 500 healthcare providers over three years and found that those who received training through the PPP initiative were significantly more likely to adhere to SCD management guidelines (OR: 2.7, 95% CI: 1.9–3.8) and provide comprehensive care to patients compared to those who did not receive training. The success of this initiative highlights the importance of capacity-building efforts in improving the quality of SCD care^[32]^. Additionally, the introduction of newborn screening programs for SCD through PPPs has been a significant milestone in early diagnosis and intervention^[29]^. A pilot project conducted by the Ministry of Health in partnership with a private diagnostics company and an international NGO introduced newborn screening in five districts in northern Uganda^[33]^. A study by Grosse *et al* evaluated the outcomes of this screening program. The study, which included a sample size of 5000 newborns, found that early diagnosis through the screening program led to a 40% reduction in mortality among SCD infants in the first year of life (OR: 0.6, 95% CI: 0.4–0.9). This initiative demonstrates the potential of PPPs in enhancing early diagnosis and improving survival rates among SCD patients^[16]^. Figure 2: PPP in SCD Management in Uganda (provided by author).

### Successful PPP models for SCD in Uganda

Uganda has witnessed several successful PPP models aimed at tackling SCD, which can offer valuable insights for other resource-limited regions. These models focus on collaboration between government entities, private healthcare providers, NGOs, and community stakeholders to improve awareness, access to treatment, and overall healthcare delivery for individuals living with SCD. Below are some key examples of successful PPPs in Uganda, highlighting their practical applications and potential for adaptation in other settings^[25]^.

## The sickle cell initiative by the Ugandan Government and Private Sector

The Ugandan Ministry of Health, in collaboration with private healthcare organizations, launched a comprehensive SCD initiative in 2017. The initiative aims to increase awareness, improve early diagnosis, and provide accessible treatment across the country. The government partnered with private health facilities to increase the availability of screening tests, including hemoglobin electrophoresis, and provide free or subsidized treatment for SCD patients. The government partnered with private media companies to run mass awareness campaigns about SCD, focusing on early detection, stigma reduction, and educating communities about the genetic nature of the disease. Private healthcare providers offered affordable diagnostic tests, such as hemoglobin electrophoresis, in collaboration with government-funded public hospitals. This helped overcome the challenge of limited diagnostic infrastructure. In partnership with NGOs, private partners provided subsidized or free treatments, such as hydroxyurea and blood transfusions, for children under the age of 5. Through the public–private diagnostic network, more individuals, particularly children, were diagnosed early, leading to better outcomes. Private hospitals and NGOs played a crucial role in filling the gaps in public healthcare delivery, particularly in urban and peri-urban areas, by offering subsidized treatments that were previously unaffordable. The initiative received strong community buy-in, largely due to the government’s collaboration with local leaders and influencers, helping reduce stigma and discrimination against SCD patients. Other regions can replicate this model by forming collaborations between ministries of health, private hospitals, diagnostic centers, and NGOs. These partnerships can target improving early diagnosis through accessible screening programs and ensure that treatment is available at affordable rates. Regions facing stigma and cultural misconceptions about SCD can adopt public awareness programs that use mass media and community influencers, similar to Uganda’s initiative, to reduce barriers to diagnosis and treatment^[^26,27^]^.

## Blood transfusion PPP model for SCD management

Given the high incidence of blood transfusions in SCD management, Uganda established a PPP model to address the shortage of safe blood. The model involves collaboration between the UBTSs, private blood banks, and local health facilities to ensure a consistent and safe supply of blood for SCD patients. The government and private hospitals organized regular blood donation campaigns, engaging the public to donate blood for individuals with SCD. These drives were promoted through partnerships with private companies and community organizations, raising awareness about the importance of blood donations for SCD management. Private hospitals and blood banks collaborated with UBTS to ensure that blood transfusions were available at reduced costs for SCD patients, particularly in emergencies. The private sector also provided technical expertise in maintaining blood safety standards. Private blood banks were integrated into the national blood inventory system, improving the reliability and distribution of blood supplies across the country. The collaboration led to an increase in the availability of blood for patients with SCD, especially during times of crisis, such as during malaria outbreaks when demand for blood spikes. The private sector’s involvement in blood screening and testing improved blood safety standards, reducing the risk of transfusion-transmitted infections. Other regions facing similar blood shortages for SCD management can adopt the model of community-based blood donation drives, supported by private companies, community leaders, and health institutions. These drives can be held regularly and strategically timed to meet peak demand periods. Regions can integrate private blood banks into the national blood system to ensure better standards of blood collection, testing, and storage. Partnerships with international organizations for training and quality assurance can further enhance this approach^[^28,29^]^.

## Collaboration with pharmaceutical companies for medication accessibility

In Uganda, private pharmaceutical companies, in collaboration with the Ministry of Health and international NGOs, have worked together to improve access to essential medications for SCD, particularly hydroxyurea, pain management drugs, and other supportive treatments. This PPP aims to lower the cost of essential drugs and ensure their consistent availability at public health centers. Private pharmaceutical companies, in partnership with the government, have negotiated bulk purchasing agreements to reduce the cost of key medications like hydroxyurea. These savings are passed on to healthcare providers, making the medications more affordable for patients. Private companies assist in the distribution of drugs to remote health centers, ensuring that SCD patients across Uganda have access to necessary medications without needing to travel long distances. NGOs and private companies also contribute to education on the importance of medication adherence for SCD patients, reducing treatment interruptions and improving patient outcomes. The subsidization of essential medications has made SCD management more affordable for many Ugandans, reducing the financial burden on families. The partnership has led to more reliable supply chains for critical medications, reducing periods of stockouts at public health facilities. Other regions with limited access to SCD medications can adopt bulk purchasing and subsidization strategies, working with both government bodies and private pharmaceutical companies to negotiate better pricing. These collaborations can be replicated in other regions by developing distribution networks that include both public and private entities, ensuring that medications are accessible even in remote areas^[^31–33^]^.

## Patient and family support networks

Uganda has established patient support groups for families living with SCD, facilitated by both private and public sector partners. These support networks provide a variety of services, including psychosocial support, financial assistance, and access to resources for managing the disease. Private health organizations, in partnership with NGOs, have established support groups that offer counseling, peer support, and educational resources for patients and their families. These groups help reduce the social isolation often felt by SCD patients. Some private sector partners provide direct financial assistance for transportation costs to healthcare centers, medical costs, and other burdens associated with long-term care for SCD. Support networks ensure that families have access to informational materials, including guidance on managing SCD at home and understanding the complexities of the disease. Families report reduced levels of stress and anxiety, as they feel supported by a community of individuals facing similar challenges. Access to financial and logistical support helps families adhere to prescribed treatment regimens, improving patient health outcomes. Other regions can establish similar peer support networks, with both public and private partners contributing resources to ensure that families receive psychosocial support, financial assistance, and access to educational resources. It’s essential to integrate not just medical care but also mental health and social support into the care pathway for SCD patients. This model can be adapted in regions with similar social and healthcare challenges^[^34–36^]^.

### Role of PPPs in SCD management

PPPs play a crucial role in addressing the multifaceted challenges posed by SCD in Uganda. Given the disease’s high prevalence, the strain it places on Uganda’s healthcare system, and the gaps in care, PPPs can offer innovative solutions to improve diagnosis, treatment, prevention, and overall management. These partnerships involve collaboration between the public sector, private companies, NGOs, and international donors, combining their respective resources, expertise, and innovations^[24]^.

### Awareness and advocacy campaigns

One of the critical areas where PPPs have made a notable impact is in the development and implementation of national awareness campaigns. These initiatives aim to educate both the general population and healthcare professionals about SCD, emphasizing the importance of early diagnosis and management^[25]^.

- Public sector role: The Ugandan government, through the Ministry of Health and the NSCCP, plays a leading role in providing education on SCD prevention, early diagnosis, and treatment. However, these efforts can often be limited by budgetary constraints and reach^[26]^.
- Private sector role: Private companies, including pharmaceutical and healthcare service providers, collaborate with the public sector and NGOs to raise awareness about SCD. For example, mobile health technologies have been used in partnership with telecom companies to distribute educational content, reaching individuals in remote areas. NGOs such as the USCRF also partner with the government to spread awareness and provide support for families affected by SCD^[27]^.

### Access to screening and early diagnosis

Early diagnosis of SCD is vital for initiating appropriate care and preventing complications. However, many regions in Uganda, particularly rural areas, face challenges in accessing diagnostic facilities and services. PPPs can bridge this gap by enhancing diagnostic infrastructure and making screening more accessible^[28]^.

- Public sector role: The Ugandan government has introduced newborn screening programs, yet these programs are often limited in scope due to resource constraints. Efforts are often concentrated in urban centers, leaving rural populations underserved.
- Private sector role: PPPs with private healthcare providers, diagnostic laboratories, and medical device companies can expand the reach of newborn screening and SCD testing. For instance, private clinics and laboratories can partner with the government to offer subsidized or free screening for children, especially in underserved regions. Mobile health units, in partnership with private medical technology companies, can travel to remote areas to provide screening services^[28]^.

### Improved access to treatment and medication

Access to proper treatment and medication for individuals with SCD, such as hydroxyurea, blood transfusions, and pain management therapies, remains a significant challenge in Uganda. PPPs can help provide more affordable and timely treatment options^[29]^.

- Public sector role: The government provides some subsidized treatments, but budgetary constraints often limit the availability of essential medications, especially for families living in poverty.
- Private sector role: Pharmaceutical companies partner with the public sector to ensure the availability of affordable medications for SCD management. For example, companies may provide discounts or collaborate with the Ministry of Health to distribute medication at lower prices or through charitable donations. Additionally, private blood banks and medical facilities can help improve access to blood transfusions, which are a critical component of SCD care^[30]^.

### Capacity building for healthcare providers

Healthcare workers, particularly in rural areas, often lack specialized training in the management of SCD, leading to suboptimal care. PPPs can play a key role in addressing these gaps by investing in training and education for healthcare professionals.

- Public sector role: The government is responsible for providing general healthcare training but may not always have the resources or capacity to offer specialized education for managing complex diseases like SCD.
- Private sector role: Through collaborations with medical schools, private healthcare providers, and international partners, the private sector can offer specialized training programs for doctors, nurses, and other healthcare professionals. This training can include updated treatment protocols, pain management techniques, and new developments in SCD therapies. Additionally, partnerships with academic institutions can promote research and the development of best practices for SCD care^[31]^.

### Research and innovation

The development of new treatments and therapies for SCD is essential for improving patient outcomes. PPPs can foster collaboration between private companies, universities, and research institutions, leading to advances in medical research and innovative healthcare solutions.

- Public sector role: The Ugandan government, through national health research institutions, plays an important role in supporting health-related research and promoting national health priorities such as SCD.
- Private sector role: Pharmaceutical companies, biotechnology firms, and academic institutions in the private sector can partner with the government to conduct research aimed at improving SCD treatment. These collaborations may focus on drug development, gene therapies, or innovations in diagnostic technologies. Additionally, partnerships with international research organizations can bring new insights and global expertise to the Ugandan context^[32]^.

### Strengthening healthcare infrastructure

Adequate healthcare infrastructure is crucial for the effective management of SCD, especially in rural and remote areas. PPPs can play a pivotal role in improving healthcare facilities and expanding the reach of care.

- Public sector role: The government is responsible for the overall healthcare system and infrastructure development, but limited resources can hinder the expansion and maintenance of healthcare facilities, particularly in rural areas.
- Private sector role: Private healthcare providers can contribute to the development of new healthcare facilities or the upgrading of existing ones. This includes building or expanding clinics and hospitals, equipping them with modern medical technologies, and ensuring they have the necessary resources to manage SCD patients effectively. Additionally, partnerships with private transport companies can improve access to care by providing patient transport services to health centers^[32]^.

### Limitations and challenges of PPPs in managing SCD in Uganda

SCD is one of Uganda’s most pressing public health challenges, with a significant impact on both the affected individuals and the healthcare system. The introduction of PPPs has provided a promising avenue for improving the diagnosis, treatment, and prevention of SCD. However, despite the potential of these partnerships, there are several limitations and challenges that need to be overcome for a more effective, sustainable solution^[33]^.

## Funding constraints and financial sustainability

In Uganda, public health funding is generally limited, and noncommunicable diseases like SCD often compete for resources with infectious diseases such as malaria and HIV. The government’s health budget has historically been underfunded, and there is a heavy reliance on donor funding, particularly from international agencies and NGOs. While this funding has supported some initiatives, it is often inconsistent and short-term. Additionally, the costs of essential SCD treatments (such as hydroxyurea, blood transfusions, and pain management) remain a significant burden for both public and private sectors. The reliance on short-term donor funds can result in the failure of SCD programs once funding is exhausted. Private companies involved in these partnerships are often reluctant to make long-term investments without assurances of a return on investment, and the absence of a clear, long-term financial strategy leaves many initiatives vulnerable to discontinuation. The inability to sustain financial resources for crucial interventions, such as medication supply chains, screening programs, and awareness campaigns, limits the overall effectiveness of PPPs^[34]^.

## Healthcare infrastructure deficiencies

Uganda’s healthcare infrastructure faces significant challenges, especially in rural areas. Public health facilities are often understaffed, under-resourced, and lack essential diagnostic and therapeutic equipment. In particular, specialized care for SCD patients – such as hemoglobin electrophoresis, bone marrow testing, and comprehensive pain management – is not readily available in many health centers. The country has a limited number of SCD treatment centers, and even fewer with specialized personnel like hematologists or genetic counselors. The private sector’s involvement in healthcare provision often focuses on high-demand, high-profit areas, which may not include specialized care for chronic conditions such as SCD. PPPs may struggle to expand their reach in such an environment. While NGOs and private entities have attempted to establish community-based programs and mobile screening units, the lack of infrastructure – especially diagnostic tools and treatment centers – hinders the scale-up of these interventions. Without significant infrastructure improvement, these partnerships cannot provide effective long-term solutions to SCD management^[35]^.

## Limited trained healthcare personnel

The healthcare workforce in Uganda is facing severe shortages of trained medical professionals, particularly in specialized fields such as hematology and genetic counseling. The shortage of skilled personnel affects the ability to diagnose, treat, and manage complex diseases like SCD. There is also a lack of continuous medical education opportunities for healthcare workers in rural or remote areas, further exacerbating this issue. Private organizations often bring expertise into PPPs, but their capacity to train and retain local staff is constrained by limited resources. Efforts to train healthcare workers in the diagnosis and management of SCD are often not adequately funded or scaled up. Additionally, the existing healthcare system is heavily reliant on a few overworked professionals, and the lack of workforce expansion means that many initiatives cannot reach the breadth of healthcare workers needed for sustainable change. PPPs that focus on capacity-building may encounter difficulties retaining trained professionals due to financial constraints and limited career development opportunities^[36]^.

## Inconsistent government policies and bureaucratic delays

While the Ugandan government has recognized the importance of addressing noncommunicable diseases, SCD has not been prioritized within national health strategies. Public health initiatives often face bureaucratic bottlenecks due to slow policy development, conflicting priorities between sectors, and lack of coordination among ministries. Although the government has made strides in expanding health insurance and including SCD in public health discussions, the lack of an integrated policy framework hampers large-scale intervention efforts. Government inefficiency in terms of policy implementation and regulation can significantly hinder the success of PPPs. PPP projects often rely on government support for approval, regulation, and financial incentives. The long wait for regulatory approval of health programs, medicines, or equipment can delay or cancel projects, reducing the willingness of private-sector participants to engage. Inconsistent policies regarding healthcare financing, insurance, and regulatory practices also create an environment of uncertainty for private partners, discouraging long-term investments^[37]^.

## Societal stigma and cultural barriers

Stigma surrounding SCD is pervasive across many parts of Uganda, as individuals with the disease are often marginalized or seen as being cursed or unlucky. This stigma can extend to family members and result in reluctance to seek medical treatment or engage in genetic counseling. Cultural misconceptions about genetic disorders, combined with limited public understanding of SCD, make it difficult for public health programs to change behaviors, particularly regarding screening and early diagnosis. PPP efforts to improve awareness and reduce stigma face significant cultural resistance. Although there have been campaigns to educate the public about SCD, cultural perceptions of the disease as a punishment or divine retribution persist. This social barrier means that even the best-planned PPP initiatives are sometimes unsuccessful in reaching affected populations. Public–private collaborations need to address these issues directly by involving local communities and leaders, ensuring culturally sensitive approaches are used in awareness campaigns, and fostering acceptance of genetic screening^[38]^.

## High cost of treatment and inaccessibility of essential medicines

SCD treatment is expensive, especially when considering the costs of medications (e.g. hydroxyurea), blood transfusions, regular check-ups, and crisis management. Uganda’s health insurance system, while improving, does not comprehensively cover the costs of long-term chronic disease management, leaving many families to bear the financial burden. Private-sector involvement in providing treatments often leads to higher costs for patients due to profit margins, even in collaborative programs. Although PPPs have worked to reduce costs through subsidies or donations, there is a mismatch between the prices of life-saving drugs and the financial capabilities of the population. Without an effective system to subsidize these costs or an expanded insurance model to cover chronic diseases like SCD, treatment remains inaccessible for a large portion of the affected population. This financial barrier often leads to patients delaying treatment, resulting in worse health outcomes and increased hospitalization costs^[39]^.

## Weak data collection and disease surveillance systems

Uganda faces significant challenges in healthcare data collection, with many health facilities lacking robust systems to track disease prevalence and outcomes. Disease registries are underdeveloped, and healthcare workers often do not have the tools or resources to collect detailed information on SCD patients. The absence of comprehensive national surveillance hinders effective planning, evaluation, and resource allocation for SCD management. PPPs that aim to implement evidence-based solutions to SCD face difficulties when there is insufficient data to guide their interventions. The lack of data means that PPPs are often working with incomplete or outdated information, making it challenging to assess the effectiveness of interventions or target the most vulnerable populations. Accurate and reliable data is essential for attracting further investment into the public and private sectors, as well as for advocacy purposes^[40]^.

## Unequal distribution of services between urban and rural areas

While Kampala and other major cities have a better distribution of healthcare facilities and services, rural areas face significant barriers in accessing even basic healthcare, let alone specialized treatment for SCD. The geographic disparity in healthcare availability exacerbates the problem of SCD in Uganda, as rural populations remain underserved by both public and private healthcare systems. PPPs are often concentrated in urban area, which leads to limited access for rural patients. Private entities, including pharmaceutical companies and NGOs, may not have the financial incentive to expand to less populated areas, and logistical challenges – such as transportation difficulties and poor road networks – further limit access. Public health initiatives in urban areas may fail to reach the rural poor who form a significant portion of SCD patients. Innovative models such as mobile clinics, telemedicine, and partnerships with community health workers could help bridge this gap, but these solutions remain underdeveloped^[38]^.

## Limited private-sector involvement in research and innovation

Despite global advancements in SCD research, including gene therapy and novel drug therapies, Uganda lacks substantial private-sector involvement in local research and development for SCD. Local pharmaceutical companies and biotechnology firms have not made significant investments in developing treatments tailored to Uganda’s specific demographic and genetic profile. The limited private-sector engagement in research slows down the development of new and affordable treatments, leaving Uganda reliant on imported, often expensive, drugs. Without a strong local research ecosystem, PPPs are restricted to implementing existing solutions rather than driving innovations that could better address Uganda’s SCD needs^[39]^.

## Lack of integration with other health programs

SCD is often treated as an isolated issue rather than integrated into broader healthcare initiatives. Public health programs in Uganda typically focus on infectious diseases, maternal health, and nutrition, leaving chronic conditions like SCD underfunded and overlooked. The lack of integration between SCD management and other public health programs limits the potential for coordinated, multi-disease interventions. PPPs in SCD may operate in silos, missing opportunities to share resources or collaborate with other sectors, such as maternal and child health programs. A more integrated approach would help streamline care, reduce duplication of efforts, and create a unified response to SCD within Uganda’s overall healthcare framework^[40]^.

### Limitations and challenges of implementing PPPs for SCD in resource-limited settings

In resource-limited settings, where healthcare infrastructure is often fragile and financial resources constrained, PPPs have emerged as a promising model for improving healthcare delivery. SCD, a hereditary blood disorder that disproportionately affects populations in sub-Saharan Africa, demands comprehensive and sustainable healthcare interventions, including early diagnosis, ongoing treatment, and patient support systems. PPPs, by leveraging the strengths of both public institutions and private entities, hold the potential to bridge critical gaps in SCD management. However, despite their theoretical benefits, the real-world implementation of PPPs in SCD care faces several substantial challenges that hinder their effectiveness and sustainability^[41]^.

### Financial constraints and the struggle for sustainability

Funding remains the backbone of any healthcare intervention, and SCD management is no exception. However, in resource-limited settings, financial limitations pose a significant hurdle to the success of PPPs. While governments in these settings often lack the fiscal capacity to fully fund SCD programs, private partners frequently require assurance of return on investment before committing resources. The high cost of managing SCD, including diagnostic testing, regular blood transfusions, and hydroxyurea therapy, further exacerbates the challenge. The reliance on donor funding, which is often short-term and project-specific, creates a cycle of dependency where initiatives start strong but struggle to maintain momentum once external support wanes. Furthermore, the private sector, particularly pharmaceutical and healthcare companies, may prioritize high-return investments such as infectious disease programs, leaving SCD – a chronic noncommunicable disease – at a funding disadvantage. Even when funding is secured, inefficiencies in fund allocation and corruption in some public institutions can limit the impact of financial investments^[^41,42^]^.

### Weak health infrastructure and service delivery gaps

In many low-income countries, health infrastructure is inadequate to support effective SCD care. Diagnostic services are often concentrated in urban tertiary healthcare centers, leaving rural populations underserved. The absence of newborn screening programs, which are crucial for early detection and intervention, further complicates the challenge. Private-sector laboratories and hospitals, which could help bridge this gap, often, charge fees that are unaffordable for the majority of the affected population. Additionally, blood transfusion services remain critically underdeveloped. Safe and consistent blood supply is an essential component of SCD management, yet many healthcare facilities in resource-limited settings struggle with blood shortages due to inadequate donation programs and poor storage facilities. While private entities can contribute to improving blood banking systems, logistical constraints and regulatory hurdles often impede their participation in public healthcare initiatives. Another major challenge is the shortage of trained healthcare professionals specializing in hematology and sickle cell care. Public healthcare systems in low-income settings frequently experience brain drain, where trained professionals seek better opportunities abroad or in private institutions, leaving public hospitals understaffed. Although PPPs could theoretically facilitate training programs, alignment of interests and long-term commitments from private stakeholders remain difficult to achieve^[^42,43^]^.

### Policy and regulatory barriers to effective PPP implementation

The success of PPPs is highly dependent on the presence of well-defined policies and regulatory frameworks. However, in many resource-limited settings, such frameworks are either weak or entirely absent. Governments may lack clear strategies for integrating private sector contributions into national healthcare plans, leading to fragmented and poorly coordinated initiatives. Bureaucratic inefficiencies further slow the implementation of PPPs. Lengthy approval processes, inconsistent regulations, and political instability contribute to delays in the rollout of essential SCD programs. Furthermore, corruption and lack of transparency in public institutions discourage private-sector involvement, as businesses are wary of engaging in partnerships where accountability is uncertain. Data availability and surveillance also remain significant concerns. Many resource-limited settings lack comprehensive epidemiological data on SCD, making it difficult to plan and allocate resources effectively. Private entities often depend on data-driven decision-making, and the absence of reliable information creates hesitancy in committing to long-term investments. Strengthening health information systems and fostering collaborative data-sharing mechanisms between public and private partners could help overcome this challenge, but implementation remains slow due to competing priorities in overburdened health ministries^[44]^.

### Equity and accessibility issues in PPP-driven healthcare models

Equitable healthcare access is a fundamental concern in SCD management, yet many PPP initiatives inadvertently exacerbate existing disparities. Private healthcare providers tend to establish facilities in urban centers, where patients are more likely to afford care, leaving rural populations with limited or no access to specialized SCD services. Even when PPPs succeed in expanding healthcare infrastructure, high out-of-pocket costs can still prevent economically disadvantaged patients from benefiting from these services. In countries where health insurance coverage is limited, the financial burden of SCD treatment remains overwhelmingly placed on families. While PPPs could play a role in supporting the development of health insurance schemes, private insurers may be reluctant to cover SCD care due to its long-term cost implications. Furthermore, patients from marginalized communities often experience additional barriers, including lack of transportation, cultural misconceptions about SCD, and stigma associated with the disease. Without deliberate efforts to design inclusive healthcare models, PPPs risk leaving behind those who need care the most^[45]^.

### Coordination and alignment of stakeholder interests

One of the fundamental challenges of PPPs in healthcare is ensuring alignment between public and private sector priorities. Public institutions focus on universal access and affordability, while private entities often prioritize financial sustainability and efficiency. In cases where these interests diverge significantly, PPPs struggle to find common ground. The lack of a shared vision frequently results in fragmented interventions, where multiple stakeholders implement parallel initiatives without effective coordination. Transparency and accountability also play a critical role in PPP success. Without clear mechanisms for monitoring and evaluation (M&E), inefficiencies and mismanagement can arise, undermining the potential impact of partnerships. A centralized coordination body within national health ministries could help streamline efforts, but implementation of such structures requires political will and sustained commitment^[46]^.

### Global implications of PPPs in managing SCD: insights for resource-limited settings

While this manuscript focuses on the specific challenges and limitations of PPPs in managing SCD in Uganda, the lessons learned from this context offer valuable insights for the broader global effort to address SCD in other resource-limited settings. These settings share many common healthcare challenges, and understanding how to effectively implement and scale PPPs in Uganda provides a roadmap for similar countries facing high burdens of SCD and other chronic diseases^[47]^.

## Contextual adaptation and scalability of PPP models

SCD affects millions worldwide, particularly in sub-Saharan Africa, parts of the Middle East, India, and the Caribbean. The challenges faced by Uganda in managing SCD – such as funding constraints, insufficient healthcare infrastructure, and limited access to treatment – are not unique. Other countries in sub-Saharan Africa, the Indian subcontinent, and parts of the Caribbean experience similar hurdles in addressing chronic diseases. Consequently, the success of PPPs in Uganda, if properly adapted, offers a model that can be replicated in other countries with similar healthcare and socioeconomic challenges. The findings from Uganda suggest that any PPP model designed to tackle SCD should be highly adaptable to local contexts. Factors like healthcare infrastructure, financial sustainability, and societal attitudes toward SCD can vary significantly across different regions, and interventions must be tailored to these variables. For example, the use of mobile clinics or telemedicine to reach rural populations could be adapted to settings like India, where rural populations face similar geographic barriers to healthcare access. Additionally, leveraging local resources, such as community health workers or mobile health apps, could enhance PPP reach in low-resource settings^[5]^.

## Financial sustainability and investment in long-term solutions

One of the significant challenges in Uganda’s PPP model for SCD is the reliance on donor funding and short-term financial commitments. This issue is common in many LMICs, where healthcare systems are underfunded and donor-driven initiatives can lack long-term sustainability. International donors play a crucial role in the healthcare systems of many resource-limited countries, but their funding often fluctuates, which makes it difficult for programs to plan long-term. The financial sustainability issue highlighted in Uganda’s experience can serve as a critical lesson for other countries with similar healthcare funding constraints. Successful PPPs in SCD management must ensure a clear strategy for financial sustainability, such as blending public and private investments, exploring innovative financing mechanisms like social impact bonds, or incorporating local health insurance models. Long-term investment in SCD treatment infrastructure – such as the development of locally sourced medications, training programs for healthcare workers, and local manufacturing of medical supplies – would reduce dependency on donor funds and increase the program’s sustainability in other settings^[3]^.

## Capacity building and workforce development

A shortage of trained healthcare professionals is a key challenge in Uganda, particularly in specialized fields like hematology, genetic counseling, and sickle cell management. This issue is not exclusive to Uganda, as many countries in Africa, South Asia, and Latin America face similar shortages of healthcare professionals, which limits the ability to deliver specialized care for chronic conditions like SCD. Other countries can draw lessons from Uganda’s struggles in workforce development. PPPs should prioritize capacity building by investing in training programs, scholarships for healthcare professionals, and long-term retention strategies, especially in underserved areas. Furthermore, public–private collaboration should focus on increasing the availability of continuing medical education and professional development to ensure that healthcare workers remain up-to-date with advancements in SCD care. International collaborations between governments, academic institutions, and private companies can help address the shortage of skilled healthcare professionals and improve access to quality care in resource-limited settings^[48]^.

## Integrating cultural sensitivity in public health campaigns

Stigma and cultural misunderstandings surrounding SCD are significant barriers in Uganda, and this challenge extends beyond Uganda’s borders. Across the world, SCD is often misunderstood, and affected individuals face discrimination and stigma, particularly in regions where genetic counseling and early diagnosis are not prioritized. Cultural perceptions of SCD as a curse, punishment, or genetic flaw remain pervasive in many settings, including sub-Saharan Africa, South Asia, and the Middle East. The importance of culturally sensitive public health campaigns cannot be overstated. PPPs aimed at improving SCD awareness and reducing stigma should consider the local cultural context. In countries where SCD stigma is prevalent, PPPs must work with local leaders, influencers, and community groups to shift perceptions about the disease. This includes engaging in dialogue about genetic disorders, addressing misconceptions, and promoting the benefits of early diagnosis and treatment. Public–private collaborations can foster community-based outreach programs that incorporate local traditions and beliefs, ultimately making awareness campaigns more effective and socially acceptable^[49]^.

## Strengthening disease surveillance and data systems

Weak disease surveillance and data collection systems are a persistent issue in Uganda and many other LMICs. Inadequate data on the prevalence and treatment outcomes of SCD makes it difficult to plan, monitor, and evaluate public health interventions. This lack of robust health data is also common in countries such as Nigeria, India, and some Caribbean nations, where fragmented healthcare systems hinder the collection and sharing of health data. Investing in reliable data collection systems should be a priority for PPPs in managing SCD. Countries facing similar data gaps can benefit from collaborative efforts to strengthen national health information systems, implement electronic health records, and establish robust disease registries. A more comprehensive data framework would allow for better-targeted interventions, improved disease monitoring, and more accurate policy-making. International collaborations can also facilitate the exchange of data and research findings, promoting best practices and informing evidence-based decision-making in SCD care^[24]^.

## Addressing geographic and socioeconomic inequities in healthcare access

Geographic barriers to healthcare are a significant challenge in Uganda, with rural populations having limited access to specialized care. This issue is echoed in countries with large rural populations, such as India, Bangladesh, and parts of sub-Saharan Africa. Access to SCD care in these regions is further compounded by poverty, limited transportation options, and the absence of healthcare infrastructure. The geographic disparity in healthcare access presents a critical challenge that PPPs must address. Lessons from Uganda’s experience suggest that innovative solutions, such as mobile clinics, telemedicine, and community-based healthcare models, can improve access to care in remote areas. Private companies, particularly those in the technology and telecommunications sectors, can play a key role in facilitating these solutions by providing the necessary infrastructure. PPPs should also consider implementing subsidized healthcare services for low-income populations to ensure that socioeconomic status does not become an insurmountable barrier to accessing SCD care^[50]^.

## Expanding access to affordable treatment and medication

The high cost of SCD treatment and medication remains a significant barrier to care in Uganda, and this issue is mirrored in other low-income settings where healthcare is either unaffordable or unavailable. Access to essential treatments like hydroxyurea, blood transfusions, and pain management remains a challenge in many LMICs, where drug costs are often prohibitively high. Affordable access to SCD treatment is a global concern. PPPs can address this issue by negotiating reduced prices for essential medications, supporting local production of SCD-related drugs, and exploring alternative treatment options that are cost-effective and suitable for resource-limited settings. Partnerships with pharmaceutical companies, local manufacturers, and governments can help drive down costs and improve accessibility. Additionally, PPPs could advocate for the inclusion of SCD in national health insurance schemes to ensure long-term coverage for patients, especially in countries where health insurance is still underdeveloped^[^51,52^]^.

## Leveraging international collaborations and global networks

SCD is a global disease that affects millions of people worldwide, and its management requires a multinational, collaborative approach. International partnerships, including collaborations with organizations like the WHO, the Global Fund, and private entities such as pharmaceutical companies, have the potential to accelerate the fight against SCD in resource-limited settings. Countries dealing with similar challenges to Uganda’s can benefit from stronger international collaborations, sharing best practices, and fostering research into affordable treatment and prevention methods. By tapping into global networks and collaborating with international stakeholders, PPPs can leverage external resources, technical expertise, and research findings to enhance SCD management. These partnerships could also create a more unified global response to SCD, making it easier for countries to adopt proven strategies for improving diagnosis, treatment, and prevention^[^53,54^]^.

### Future directions for strengthening PPPs in the management of SCD in Uganda

SCD remains a critical health challenge in Uganda, where the prevalence of the disease is notably high. In this context, PPPs have shown potential in enhancing the management and care of SCD patients. However, while PPPs have demonstrated promise in bridging gaps in healthcare delivery, there is still significant room for improvement. In order to effectively address the needs of SCD patients and ensure sustainable care, several future directions must be considered for strengthening PPPs in Uganda. These directions will address both systemic challenges and the strategic engagement of all stakeholders involved^[36]^.

## Expanding and enhancing public–private collaboration

One of the most significant steps in strengthening PPPs for the management of SCD is expanding the collaboration between the public and private sectors. While some initiatives have already shown success, scaling these efforts requires deeper engagement from both sides of the partnership. Future PPPs should be guided by a more integrated policy framework that ensures alignment between national health priorities and private sector interests. The government needs to engage the private sector through clearly defined policies that not only incentivize participation but also ensure alignment with public health goals. Strengthening policy frameworks will encourage private entities to invest in SCD research, healthcare infrastructure, and treatment programs that are both beneficial to their bottom line and to the public health system. A greater emphasis on formalized agreements and contracts is needed to ensure long-term commitment and sustainability of PPPs. These agreements should specify roles, responsibilities, and the financial contributions of each party, and include clear measures for M&E. Such formal arrangements can address potential ambiguities in governance, which have sometimes led to poor outcomes^[37]^.

## Increasing investment in SCD research and treatment infrastructure

Investment in both research and treatment infrastructure is crucial for improving the management of SCD through PPPs. In Uganda, many healthcare facilities lack the necessary infrastructure to provide comprehensive care for SCD patients, particularly in rural and underserved areas. Investing in SCD research is essential for developing new and more effective treatments for the disease. The private sector, with its access to capital and technological expertise, can partner with public institutions to fund research on novel therapies, diagnostic tools, and interventions. Public–private collaboration in the research space can lead to the development of tailored therapies that are affordable and effective in the Ugandan context. Additionally, expanding the scope of research to include genetic studies could aid in better understanding the genetic factors affecting SCD in the Ugandan population. Building or upgrading healthcare facilities capable of providing specialized care for SCD patients is critical. PPPs can be instrumental in financing the development of blood transfusion centers, pain management units, and comprehensive clinics that offer care for the complications of SCD. Private investors could work alongside the government to fund infrastructure projects, such as the establishment of dedicated SCD care centers in regional hospitals or clinics, thereby reducing the burden on central hospitals^[37]^.

## Improving access to SCD diagnosis and care

Access to early diagnosis and ongoing care is often limited by geographical, economic, and social barriers. PPPs can help overcome these challenges by leveraging private sector resources to enhance access to healthcare services across Uganda. To improve early diagnosis of SCD, PPPs could focus on establishing community-based screening programs. These initiatives would involve collaboration between private health organizations, local governments, and community leaders to identify individuals at risk for SCD. By integrating mobile clinics and outreach programs in underserved areas, PPPs can ensure that SCD screening becomes more accessible, especially in rural settings where healthcare infrastructure is limited. Leveraging telemedicine and digital health platforms is another avenue for improving access to SCD care. Public–private collaborations in the development and implementation of telemedicine services can enable remote consultations for patients in rural areas, reducing the need for travel and improving access to specialized care. Telemedicine can also facilitate continuous monitoring of patients’ conditions, allowing healthcare providers to intervene early in the case of complications. This approach can be particularly valuable for patients with chronic SCD who require regular follow-up^[14]^.

## Capacity building for healthcare professionals

There is an urgent need to enhance the capacity of healthcare professionals in Uganda to manage SCD effectively. Training and skill development programs, delivered through PPPs, can help ensure that healthcare workers are equipped with the knowledge and tools needed to provide the best possible care for SCD patients. PPPs can establish specialized training programs for healthcare workers, focusing on the management of SCD. These programs should target medical professionals, nurses, and laboratory technicians who play key roles in the diagnosis and treatment of the disease. Partnerships with academic institutions and international organizations can provide technical support in the design of these training curricula. In addition, ongoing professional development opportunities will ensure that healthcare providers stay up-to-date with the latest research and treatment protocols. Pain management and palliative care are essential components of SCD management. Given the chronic and debilitating nature of the disease, healthcare providers must be trained in pain management strategies, including pharmacological and non-pharmacological interventions. PPPs can help by establishing partnerships with organizations that specialize in palliative care to provide training to healthcare workers^[11]^.

## Strengthening public awareness and patient education

Increasing public awareness and patient education about SCD is essential for improving health outcomes. PPPs can play a pivotal role in developing and implementing public education campaigns that focus on disease prevention, early diagnosis, and the importance of ongoing care. The use of media campaigns, particularly through radio, television, and social media platforms, can help raise awareness of SCD and its impact on individuals and families. Private sector organizations, including media companies and mobile network providers, can partner with the government and healthcare institutions to promote awareness campaigns. These campaigns should focus on educating the public about genetic counseling, the importance of early screening, and available healthcare resources. PPPs could also focus on patient support initiatives that aim to empower individuals living with SCD. These could include peer support groups, educational materials, and outreach services that help patients manage their disease more effectively. Additionally, providing patients with access to genetic counseling and information about reproductive health can improve the quality of life for individuals with SCD^[12]^.

## Monitoring, evaluation, and sustainability

For PPPs to be successful in the long term, robust M&E frameworks need to be put in place. These frameworks will ensure that the impact of PPPs on SCD management is continuously assessed and improvements are made. PPPs should prioritize the collection of data to measure the effectiveness of interventions, track health outcomes, and identify areas for improvement. Regular evaluation of PPP initiatives can provide evidence of their impact on patient outcomes, healthcare access, and overall public health. Ensuring the long-term sustainability of PPPs is crucial. Partnerships must focus on creating financially sustainable models that do not overly rely on external funding. Private sector entities, such as insurance companies, could explore models that provide affordable health coverage for SCD patients, reducing the financial burden on individuals and families^[13]^.

## Conclusion

PPPs hold significant potential for addressing the complex challenges of SCD management in Uganda. As demonstrated by successful initiatives, PPPs can leverage the strengths of both public and private sectors to enhance healthcare delivery, expand access to essential services, and drive innovation in treatment and care. However, the effectiveness and sustainability of these partnerships are currently hindered by various challenges, including dependence on external funding, regulatory gaps, inequitable access to services, and capacity constraints. To overcome these challenges and maximize the impact of PPPs, it is crucial to focus on future directions that emphasize sustainable funding, stronger regulatory frameworks, equitable service delivery, and robust community engagement. Developing clear guidelines for PPP formation and operation, enhancing technical capacity, improving coordination, and fostering a culture of innovation are all essential steps towards building more resilient and effective partnerships. Additionally, integrating PPPs into national health strategies and securing government support will be vital for ensuring that these collaborations are aligned with broader health goals and receive the necessary resources and backing.

## Data Availability

Not applicable.

## Research registration unique identifying number (UIN)

Not applicable as this is a review.
